# Medicinal Plants Used for Neuropsychiatric Disorders Treatment in the Hauts Bassins Region of Burkina Faso

**DOI:** 10.3390/medicines4020032

**Published:** 2017-05-19

**Authors:** Prosper T. Kinda, Patrice Zerbo, Samson Guenné, Moussa Compaoré, Alin Ciobica, Martin Kiendrebeogo

**Affiliations:** 1 Laboratoire de Biochimie et Chimie Appliquées, Université Ouaga I-Pr Joseph KI-ZERBO, 03 PB 7021 Ouagadougou 03, Burkina Faso; pros.kinda@hotmail.fr (P.T.K.); guesams@gmail.com (S.G.); mcompaore_3@yahoo.fr (M.C.); 2 Laboratoire de Biologie et écologie végétale, Université Ouaga I-Pr Joseph KI-ZERBO, 03 BP 7021 Ouagadougou 03, Burkina Faso; patzerbo@yahoo.fr; 3 “Alexandru Ioan Cuza” University of Iasi, Faculty of Biology, Department of Research, Carol I Avenue, No. 20A, Iasi 700505, Romania; alin.ciobica@uaic.ro

**Keywords:** Neuropsychiatry, phytotherapy, traditional healers, Burkina Faso

## Abstract

**Background:** In Burkina Faso, phytotherapy is the main medical alternative used by populations to manage various diseases that affect the nervous system. The aim of the present study was to report medicinal plants with psychoactive properties used to treat neuropsychiatric disorders in the Hauts Bassins region, in the western zone of Burkina Faso. **Methods:** Through an ethnobotanical survey using structured questionnaire, 53 traditional healers (TH) were interviewed about neuropsychiatric disorders, medicinal plants and medical practices used to treat them. The survey was carried out over a period of three months. **Results:** The results report 66 plant species used to treat neuropsychiatric pathologies. Roots (36.2%) and leaves (29%) were the main plant parts used. Alone or associated, these parts were used to prepare drugs using mainly the decoction and the trituration methods. Remedies were administered via drink, fumigation and external applications. **Conclusions:** It appears from this study a real knowledge of neuropsychiatric disorders in the traditional medicine of Hauts Bassins area. The therapeutic remedies suggested in this work are a real interest in the fight against psychiatric and neurological diseases. In the future, identified plants could be used for searching antipsychotic or neuroprotective compounds.

## 1. Introduction

Nowadays, medicinal plant use in traditional therapy is increasing and diversifying. These plants were a precious patrimony for the humanity in general and particularly very important for developing countries people’s healthcare and their subsistence [1]. They are invaluable resources for the great majority of rural populations in Africa, where more than 80% use them to ensure their primary healthcare [2]. According to the World Health Organization (WHO), neuropsychiatric disorders are a whole of “mental health problems”, which are characterized by anomalies of the thought, emotions, behavior and relationship with others. These pathologies handicap the person concerned and assign people of its circle. Factors causing these disorders are essentially genetic, social, environmental and psychotropic drugs. Mental and neurological disorders represent 13% of the burden of total morbidity in the world [3]. Thirteen per cent to 49% of the world’s populations develop neuropsychiatric disorders at some point in their life [4]. These pathologies affect all categories of person, race, sex and age [5]. Epilepsy is one of the most common neurological disorders. It affects more than 50 million persons in the world including 80% in developing countries [6]. High prevalence was observed in Africa where about 75% of patients do not receive adequate treatment [7]. The prejudices that surround neuropsychiatric diseases are causes of stigmatization of unwell persons who are often marginalized [3,8]. In Burkina Faso, 175‰ of the cases of disability are caused by neuropsychiatric disorders [6]. 

Many natural or synthetic psychoactive molecules such as neuroleptics, antidepressants, anxiolytics are used in modern medicine to treat these pathologies, particularly epilepsy, schizophrenia and the others psychotic disorders [8,9,10]. However, these modern treatments are expensive, complex and inaccessible for African populations in rural area [8,11]. Many of these psychoactive molecules have plant origins [12,13], which could justify plants use in the African traditional medicine to treat neuropsychiatric diseases [14,15]. In Burkina Faso, medicinal plants are widely used by peoples. Disapproved a long time after independences period for allopathic drugs [16], the government allowed in 1994 the traditional medicine practice. Since this time, it appeared a craze more and more growing for phytotherapy within the population, already predisposed to be directed there [17]. Moreover, many studies were undertaken to document plant species used in this therapy practice [18,19,20,21,22]. However, little research has approached the specific case of plants used to treat nervous system disorders in Burkina Faso. In the Hauts Bassins region, these pathologies were frequently denoted in psychiatric consultation [23,24]. Except Millogo’s group works on “epilepsy and traditional medicine in Bobo-Dioulasso” [25], the traditional therapy of these pathologies is quoted only in other parallel studies. The present study aims to provide information about medicinal plants used to treat neuropsychiatric disorders in the Hauts Bassins region of Burkina Faso. It was necessary to report psychic and neurological disorders treated by traditional healers, medicinal plants and medical practices used for these treatments. 

## 2. Materials and Methods 

### 2.1. Study Area

**Figure 1:**
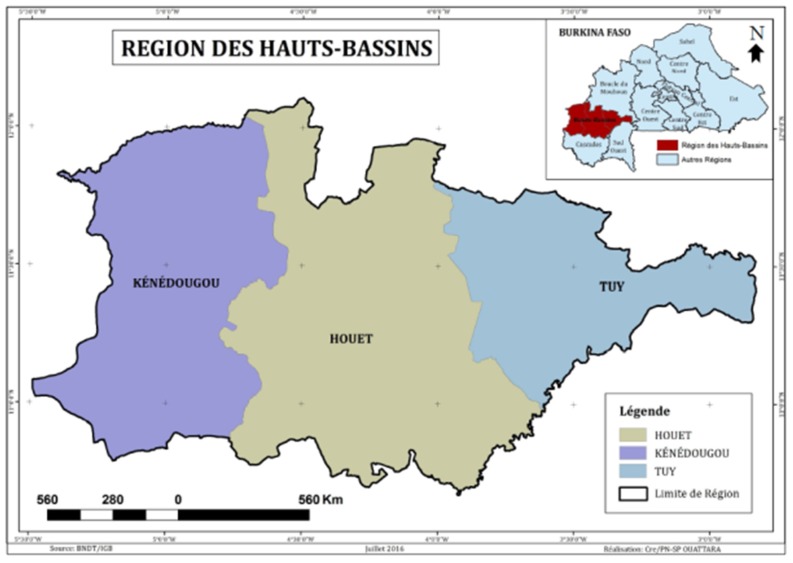
Study area localization (Hauts Bassins region of Burkina Faso).

The study was carried out in the Hauts Bassins region, located in western part of Burkina Faso (Figure 1). This area is known for its high phytogenetical and cultural diversity. Located at the West of Burkina Faso, between 9°21’N latitude and 2°27’W longitude, the Hauts Bassins region belongs to the phytogeographical sector of south-soudanien, characterized by average annual precipitations higher than 900 millimeters and average temperatures oscillating between 25 °C and 30 °C [26]. This sector is dominated by vegetable formations of savannas type timbered, arboreous or shrubby [27]. Several ethnics groups live in this area with a great diversity of cultural practices. The main spoken languages are Mooré (29.5%), Dioula (27.1%) and Bobo (18.8%) [28]. This region is characterized by a high number of traditional healers (TH) resulting from various ethnic groups. In addition to plant diversity and neuropsychiatric diseases frequency [24], the area was chosen because of the presence of various TH.

### 2.2. Ethnobotanical Data Collection 

The ethnobotanical survey was carried out during a three month period from October to December 2015. Data were collected using a structured interview with traditional healers (TH) who are organized in association. Through the association, a preliminary phone call was had with TH to inform them about objectives of the study. After that, an appointment were fixed with each one for individual interview. The approach was based on a dialogue using one of the three languages (Mooré, Dioula or French) to the TH choice. Pre-established questionnaires were used and a local person acting as a guide was necessary. Data were collected and transcribed on survey card-guides. It concerned medicinal plants used to treat the main psychiatric and neurological diseases such as epilepsy, mental disorders or madness, evils related to charm or witchcraft, hallucination or consciousness loss. These pathologies were reported to be more frequent in this area of Burkina Faso [24]. We gathered some of them because of their names in the local languages. Other collected information related to local names (in Mooré and/or Dioula) of plants, organs used of plants and medical practices such as drugs preparation and administration methods. Fifty-three TH including 35 men and 18 women, old from 31 to 82 years and having experience of plants use in traditional medicine were interviewed. Plants mentioned in the interview were collected in order to make the herbal constitution.

### 2.3. Data Analysis

Samples of plants collected were identified by botanists of the Ecology Department of University of Ouaga I-Pr Joseph Ki Zerbo (Burkina Faso). Then, voucher specimens were deposited in the herbarium of this University. The adopted nomenclature is that of “the tropical flora of Western Africa” [29], “medicinal plants and traditional medical practices in Burkina Faso” [30], “the catalogue of vascular plants of Burkina Faso” [31] and some enumerations of tropical Africa plants [32,33,34]. Plant parts used and medical practices were listed. Data were analyzed using SPSS software version 17.0 for window (SPSS Inc., Chicago, USA), and graphs were made on Excel of Office 2013.

## 3. Results

### 3.1. Plants Species Used

Sixty-six plant species including 51 woody and 15 herbaceous used to treat psychiatric and neurological diseases were identified. They belonged to 56 genera and 32 families (Table 1). Acacia and Ficus Genera were the most represented with 4 species each. The most represented families were Mimosaceae (8 species), Fabaceae (5 species) and Rubiaceae (5 species). Among these plants, the most used were showed on Table 2. A high use of Securidaca longepedunculata (45.3%), Calotropis procera (20.75%), Khaya senegalensis (20.75%), Allium sativum (20.75%), Daniellia oliveri (19%) and Annona senegalensis (17%) was observed by the majority of traditional healers (TH). Datura innoxia and Zanthoxlum zanthoxyloïdes were used by the oldest TH (more than 60 year old). Six species: S. longepedunculata, C. procera, K. senegalensis, A. senegalensis, Diospyros mespiliformis and Guiera Senegalensis were used to treat the main diseases targeted. Most of the plants were used alone and in association with other plants.

### 3.2. Plant Parts Used and Medical Practices 

**Figure 2:**
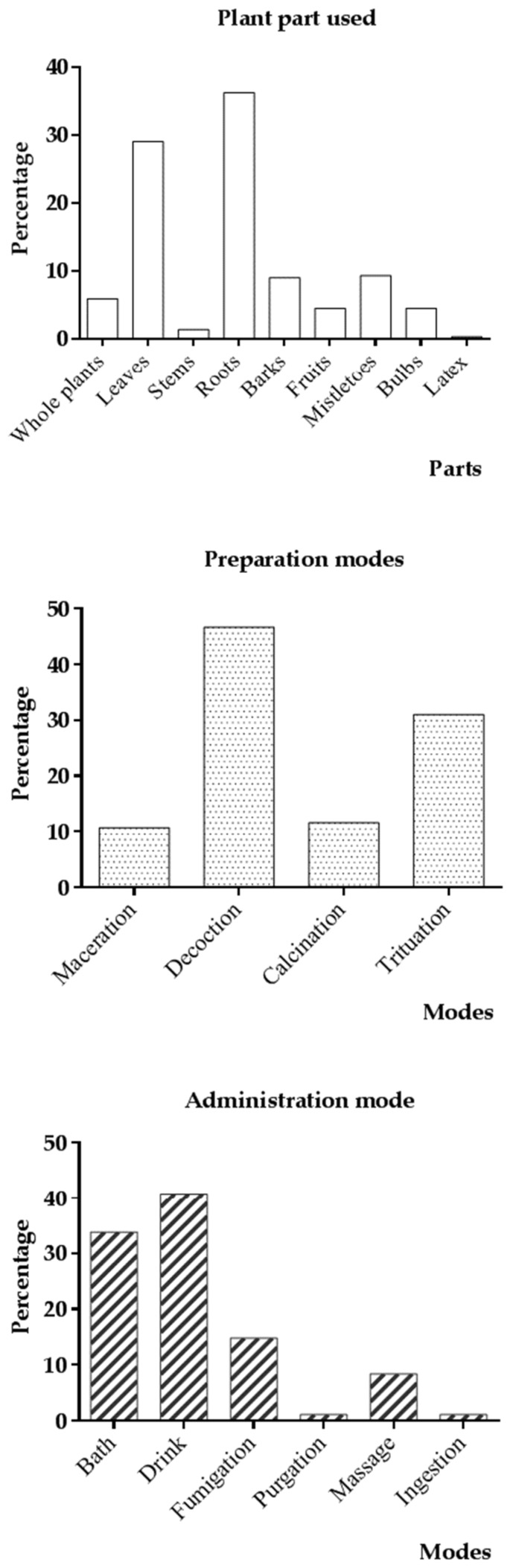
Plant parts used, modes of preparation and administration of remedies.

Various plant parts were used to prepare remedies (Figure 2a). Roots were mainly used (36.2%), followed by leaves (29%), mistletoes (9.3%) and stem barks (9%). Drugs preparation modes were the decoction (46.7%), the trituration (31%), the calcination (11.6%) and the aqueous maceration (10.7%) (Figure 2b). The drink (40.8%), the bath (33.8%), the fumigation (14.8%) and the massage (8.4%) are the main modes of administration (Figure 2c).

### 3.3. Neuropsychiatric Pathologies Treated

Diseases or regrouping diseases treated by traditional healers were registered in Table 3. From these results, hallucination or consciousness loss were most treated, followed by epilepsy, mental disorders and witchcraft or evils related to charm. In addition to these target pathologies, other cases such as insomnia and nerves diseases are also treated. Several plant species intervene in the treatment of each listed disorders. Thus, 37 plants were used to treat hallucination or consciousness loss, 32 to treat mental disorders, 31 to fight against epilepsy and 25 against diseases related to charm or witchcraft.

## 4. Discussion

Traditional medicine practice in Hauts Bassins area is rich and diversified. The most often treated neuropsychiatric disorders are hallucination, epilepsy and mental disorders, respectively treated by 79.2%, 49% and 47.2% of traditional healers (TH). These data correspond to those of other works [7,25,35], which revealed that these pathologies are well-known and treated in the traditional medicine of many African countries.

Sixty-six (66) plant species belonging to various families used in the treatment of neuropsychiatric disorders were listed. This result testifies TH knowledge about plants diversity of this area and their therapeutic virtues. Similar results were observed by previous studies [19,21] which showed that local populations of Burkina Faso were known to profit from the best part of biodiversity in traditional medicine. More than 77% of plants identified are ligneous. This rate could be justified by the relative abundance of these species in the phytogeographical sector of this area, and their availability during all the year. These results were in the same order with those of Traoré’s group in the province of Comoé [36], Olivier’s group on “Dozo” traditional healers [21] and Zerbo’s group in western area [22], which indicates a prevalence of ligneous use in the pharmacopeia of this zone of Burkina Faso. *S. longepedunculata*, *C. procera*, *K. senegalensis*, *A. sativum*, *D. oliveri*, *A. senegalensis* were identified as the main species used and *D. innoxia*, *Z. zanthoxyloïdes* were only used by older TH. They were cited like plant species intervening in the treatment of neuropsychiatric disorders in others African countries [8,35,37]. According to many authors, all these plants have phytochemical components with effects on the nervous system [38,39]. They contain alkaloids, terpenoids, steroids, flavonoids, tannins, saponins and cardiac glycosides (Table 4). These chemical constituents were considered as the main bioactive compounds of medicinal plants [30,40,41]. *C. procera* root bark used in the treatment of anxiety, epilepsy, and madness contain alkaloids such as α-amyrin, β-amyrin, while its leaf and its latex possess cardenolides such as calactin, calotoxin, calotropin and uscharin [42,43]. 

These chemical contents could be responsible of the traditional use of this plant. Besides, *C. procera* extracts were reported to possess significant anticonvulsant and analgesic properties [42,44]. Tropanic alkaloids as scopolamin, atropin, hyoscianin isolated in *D. innoxia* are known for their anticholinergic effects. They act as acetylcholin antagonists [15]. Scopolamine is an antimuscarinic agent used as analgesic and relaxant [45]. Anticholinergic and antimuscarinic effect of these compounds could explain in part Datura use in mental diseases treatement. Securidine, an alkaloid isolated from *S. longepedunculata* root, has a stimulating effect on the spinal cord. Used in a non-toxic dose, it influenced the function of the autonomic nervous system [46]. Some flavonoids were reported to possess anxiolytic effects and neuroprotective activities; they are capable of binding to GABAA receptors with significant affinity [47]. As examples, 6-methylapigenin is a benzodiazepine binding site ligand and 2S(-)-hesperidin has sedative and sleep-enhancing properties [48]. Quercetin significantly decreased the brain ischemic lesion [49]. Hesperidin was identified in *C. aurantifolia* and *Z. zanthoxyloïdes*, while apigenin was isolated from *S. longepedunculata* and Quercetin in most of plants listed in this study (Table 4).

These bioactive compounds could explain plants efficacy in the treatment of neuropsychiatric diseases [50,51]. Mechanisms through which these compounds act on the central nervous system are various including regulation of neurotransmitters activity [52,53,54]. However, benefical activities of these plants do not occult their toxic effects. Indeed, they have also cytotoxic and cardiotoxic effects [42]. Securinine in the range 5–30 g/kg act like strychnine, causing spasms and death by respiratory arrest [46]. Tropanic alkaloids are potential neurotoxic agents [15]. Therefore, a controlled use of these plants should be promoted.

Roots (36.2%) and leaves (29%) were the most used organs for the preparation of remedies. These data are in agreement with those observed by Olivier’s group [21] and Kantati’s group [35]. That would be explained by the availability of these plant parts at all periods in this region, but their effectiveness would be related to the significant accumulation of chemical compounds in these organs [140,113]. However, roots use should lead to some species disappearance. Thus, conservation measures of those are necessary.

Methods of remedies preparation are similar to those observed in other works. The decoction (46.7%) was the most used, followed by the trituration, calcination and aqueous maceration. These results are comparable to those of Zerbo’s group works in Sanan’s region and Western area of Burkina Faso [16,22], Adetutu’s group in the South-western of Nigeria [141] and Kantati’s group in Togo [35]. They noted that these methods were the main ones used by traditional healers in these different areas. In phytochemistry, the decoction is considered to be a method allowing complete extraction of bioactive chemical compounds of plants [142]. The aqueous maceration was quoted as being a good method of alkaloids and polyphenols extraction [142,143]. Likewise, the trituration and the calcination methods allow reducing vegetable material to powder or paste, while preserving bioactive molecules. These data could justify the main use of these modes of preparation.

The majority of drugs are administrated orally (drink, 40.8%), the preferential mode of administration in the traditional medicine [67]. However, some are preferentially used by external ways. That would be related to risks that oral use presents for some plants, because of their toxicity or the specificity of the disease [21]. The nasal way is the third most used mode of administration. It has the advantage of allowing a fast access of the active substances in the brain and their best absorption [144].

Results of the ethnobotanical survey corroborate with previous phytochemical studies about traditional uses of plants listed [7,35] and their psychoactive compounds content [69,91]. Indeed, alkaloids are the most known of molecules possessing psychoactive properties [67,145]. Likewise, some flavonoids, steroids and terpenoids were quoted to have psychoactive effect [47,53,146]. These chemical constituents intervene to disturb neurotransmitters activities. They stimulate, inhibit or block liberation, reception or elimination of neurotransmitters [147,148]. Pharmacological results show that the main plants used possess anticonvulsant, anxiolytic, antispasmodic, antinociceptive, analgesic or sedative properties [44,85,111]. This result could confirm the presence of psychoactive compounds in these plants.

## 5. Conclusions 

This study made it possible to report 66 plant species belonging to 51 genera and 32 families used for the treatment of neuropsychiatric diseases. Roots and leaves were the most organs used, the decoction and the trituration were the principal modes of drug preparation. The administration of remedies was done mainly by oral way. Plants identified were quoted to possess psychoactive properties and some chemical contents which could justify that.

Traditional remedies suggested in this study are a real interest in the fight against neuropsychiatric disorders. Then, further researches will be necessary to identify psychoactive compounds from these plants and their acting mechanisms for neuropsychiatric diseases treatment.

## Figures and Tables

**Table 1:** Global information on plants used in various treatments.

Scientific Name (Genera and Specie)	Family	Local Name (Mooré)	Local Name (Dioula)	Parts Used	Mode of Preparation	Mode of Administration	Pathologies Treated
Abrus precatorius L.	Fabaceae	Noraog-nini	Noronha	Fr	Cal	Mas	MA-MD
Acacia ataxacantha DC.	Mimosaceae	Kanguin pèelga		Ro, Ba	Dec	Bat, Dri	EP
Acacia nilotica (L.) Willd. Ex Del.	Mimosaceae	Peg-nenga	Bangana	Ro	Dec	Bat, Dri	MA-MD
Acacia pennata (L.) Willd	Mimosaceae	Kanguinga		Ro	Dec	Bat, Dri	EP
Acacia sieberiana DC.	Mimosaceae	Gor-ponsego	Wenekassango	Le, Ro, Ba	Mac, Dec, Tri	Bat, Dri, Fum, Mas	MA-MD, HA-CL
Adansonia digitata L.	Bombacaceae	Tohèga	Sira-yiri	Le, Ro	Dec, Cal	Bat, Dri, Fum	MA-MD, HA-CL
Afzelia africana Smith ex Pers.	Caesalpiniaceae	Kankalga	Lingué, Lingué yiri	Le, Ro, Ba, Mi	Dec, Cal, Tri	Bat, Dri, Fum	MA-MD, HA-CL
Allium cepa L.	Liliaceae	Zéyon	Djaba	Bu	Tri	Fum	EP
Allium sativum L.	Liliaceae	Layi		Bu	Dec, Cal, Tri	Bat, Dri, Fum, Pur, Mas	MA-MD, HA-CL
Annona senegalensis Pers.	Annonaceae	Barkudga	Mandé sunsun, Barkandé	Wp, Le, Ro, Ba	Dec, Cal, Tri	Bat, Dri, Fum, Mas	EP, MA-MD, CH-WI, HA-CL
Anogeissus leiocarpus (DC) Guill. & Perr.	Combretaceae	Siiga		Ba	Mac	Bat, Dri	HA-CL
Balanites aegyptiaca L.	Balanitaceae	Kyeguelga	Zèguenè	Le, Ro	Dec	Bat, Dri	MA-MD, CH-WI
Boscia senegalensis (Pers) Lam. ex Poir.	Capparidaceae	Lambwetga	Bere	Le, Ro	Dec	Bat, Dri	EP
Boswellia dalzielii Hutch	Burseraceae	Gondregneogo, Kondregneogo		Ro, Ba	Mac, Dec, Tri	Bat, Dri, Fum	HA-CL
Calotropis procera (Ait) Ait. F.	Asclepiadaceae	Putrepuuga	Fogofogo	Wp, Le, Ro, Mi, La	Mac, Dec, Tri	Bat, Dri, Fum, Ing	EP, MA-MD, CH-WI, HA-CL
Ceiba pentandra (L.) Gaertn	Bombacaceae	Gounga	Bana-yiri	Ro	Cal	Dri	EP
Cissus quadrangularis L.	Vitaceae	Wob-Zanré	Oulouyoroko	St	Cal	Dri	EP
Citrus aurantifolia (Christm.) Swingle	Rutaceae	Lembur-tiiga	Laimbourou	Fr, Mi	Mac, Dec, Cal	Bat, Dri, Mas	MA-MD, CH-WI, HA-CL
Crateva adansonii DC.	Capparidaceae	Kalguem-tohèga		Le	Dec	Dri	CH-WI
Cymbopogon giganteus Chiov.	Poaceae	Kuwaré	Tiékala	Le, Ro	Dec	Bat, Dri	MA-MD, HA-CL
Cymbopogon proximus (Hochst ex A. Rich) Stapf	Poaceae	Soompiiga		Wp, Ro	Cal	Dri	CH-WI
Dalbergia melanoxylon Guill. & Perr.	Fabaceae	Guirdiandéga		Ro	Mac	Fum	MA-MD, HA-CL
Daniellia oliveri (Rolfe) Hutch et Dalz	Caesalpiniaceae	Aoga, Anwga	sana, sana yiri	Le, Ro, Ba, Mi	Mac, Dec, Cal, Tri	Bat, Dri, Fum	MA-MD, CH-WI, HA-CL
Datura innoxia Mill.	Solanaceae	Barassé, Zèèbla	Alomoukaïkaï	Le, Fr	Cal	Dri, Mas	MA-MD,CH-WI, HA-CL,IN,ND
Detarium microcarpum Guill. & Perr.	Caesalpiniaceae	kagadéga	Tamakouma	Le, Ro	Dec	Bat, Dri	EP, CH-WI, HA-CL
*Diospyros mespiliformis* Hochst ex A. DC	Ebenaceae	Gaaka, Gaanka	Sounsoun, Sounsounfi	Le, Ro	Mac, Dec	Bat, Dri	EP, MA-MD, CH-WI, HA-CL
*Entada africana* Guill. & Perr.	Mimosaceae	Séonego		Ro	Dec	Bat, Dri	EP
*Faidherbia albida* (Del.) A. Chev.	Mimosaceae	Zaanga	Balanzan, Balãzã	Le, Ro	Dec	Bat, Dri	CH-WI
*Ficus ingens* (Miq.) Miq.	Moraceae	Kunkwiga		Ro	Dec	Dri	EP
*Ficus iteophylla* Miq.	Moraceae	Kunkwi-pèelga	Djetigui faaga, Diatiguifaga	Le, Ro, Ba	Mac, Dec	Bat, Dri	EP, MA-MD, HA-CL
*Ficus sycomorus* L.	Moraceae	Kankanga	Toro, toro yiri	Le, Ro, Mi	Dec, Tri	Bat, Dri	EP, HA-CL
*Ficus vallis-choudae* Delile	Moraceae		Torossaba, Toroba	Le	Dec	Bat, Dri	EP
*Flueggea virosa* (Roxb ex. Willd) Voigt.	Euphorbiaceae	Sugdin-daaga	Balabala, Bala-bala	Le, Ro	Dec	Bat, Dri	CH-WI
*Gardenia sp.*	Rubiaceae	Subudga, Lambrezunga	Bure, Buré yiri	Wp, Le, St, Ro	Dec, Cal	Bat, Dri	EP, CH-WI, HA-CL
Guiera senegalensis J.F. Gmel	Combretaceae	Wilin-wiiga	Koungouè, Kungouè	Wp, Le, Ro, Mi	Dec, Cal, Tri	Bat, Dri	EP, MA-MD, CH-WI, HA-CL
Hygrophila senegalensis (Nees) T. Anderson	Acanthaceae		Kelebetokala, Klebato-yiri	Le	Mac, Dec	Bat, Dri	EP
Hyptis spicigera Lam.	Lamiaceae	Rung-rungui	Timitimini.	Wp	Dec	Bat, Dri	EP
Indigofera tinctoria L.	Fabaceae	Garga		Le	Tri	Pur	HA-CL
Khaya senegalensis (Desr) A. Juss	Meliaceae	Kuka	Diala, Djala	Le, Ba, Mi	Mac, Dec, Cal, Tri	Bat, Dri, Fum	EP, MA-MD, CH-WI, HA-CL
Lannea acida A. Rich	Anacardiaceae	Labtulga		Le, Ro, Ba	Dec, Tri	Bat, Dri	EP, HA-CL
Leptadenia hastata (Pers.) Decne	Asclepiadaceae	Lelongo	Kosafla	Wp, Le, St, Ro	Dec	Bat, Dri	MA-MD, HA-CL
Mitracarpus villosus (SW.) DC.	Rubiaceae	Yod-pèelga		Wp	Tri	Fum	MA-MD
Mitragyna inermis (Willd) O. Ktze	Rubiaceae	Yiilga	Djou, Diou, Jun, dioum	Wp, Le, Ro	Dec, Tri	Bat, Dri, Fum	EP, MA-MD, HA-CL
Moringa oleifera Lam.	Moringaceae	Arzan-tiiga	Masa yiri	Ro	Dec	Bat, Dri, Fum	MA-MD
Nicotiana rustica L.	Solanaceae	Kinkirs taba, Waamb-tabré	Flavourou	Le	Tri	Fum	HA-CL
Nicotina tabacum L.	Solanaceae	Taba	Kotaba	Le	Cal	Dri, Mas	CH-WI
Ocimum americanum L.	Lamiaceae	Yulin-gnu-raaga	Sukuola	Wp, Le	Dec, Tri	Bat, Fum	EP, HA-CL
Ocimum basilicum L.	Lamiaceae	Yulin-gnuuga	Sukuola-sina	Le	Tri	Fum	HA-CL
Parkia biglobosa (Jacq.) R. BR. ex G. Don. F	Mimosaceae	Roaaga	Nèrè	Le, Ro, Mi	Mac, Dec	Bat, Dri	EP, MA-MD, CH-WI
Pennisetum americanum Stapf	Poaceae	Kazui	Sagnon	Fr	Tri	Pur	EP, CH-WI
Pericopsis laxiflora (BentH ex Bak.) V. Meeawen	Fabaceae	Taankoniliga,	Kolo-kolo, Kolokolo yiri	Le, St	Dec, Tri	Bat, Dri, Fum	MA-MD, HA-CL
Prosopis africana (Guill. Perr. & Rich) Taub.	Mimosaceae	Duanduanga, yamagui	Goulé, Gouélé	Ro, Fr	Dec, Cal	Bat, Dri	CH-WI
Pseudocedrela kotschyi (Schweinf.) Harms	Meliaceae	Siguédré		Le	Dec	Bat, Dri	MA-MD, HA-CL
Saba senegalensis (A. DC) Pichon	Apocynaceae	wèdga	Zaban yiri	Ro	Dec	Bat	HA-CL
Sclerocarya birrea (A. Rich) Hochst	Anacardiaceae	noabga		Le, Ba	Dec	Dri	EP
Scoparia dulcis L.	Scrophulariaceae	Kafremaandé		Wp	Tri	Fum	MA-MD, HA-CL
Securidaca longepedunculata Fresen	Polygalaceae	Pèlga	Djoro, Diouro	Le, Ro, Ba	Mac, Dec, Cal, Tri	Bat, Dri, Fum, Pur, Mas	EP, MA-MD, CH-WI, HA-CL
Sterculia setigera Del.	Sterculiaceae	Ponsemporgo, Putermuka	Congo-sera, Kongossira	Ro, Mi	Dec	Bat, Dri	EP
Strychnos spinosa Lam.	Loganiaceae	Katrepoaga, Katerpoagha	Kogobaranie, Fouflé barani	Fr	Tri	Ing	CH-WI
Stylosanthes erecta P. Beauv.	Fabaceae	Sakwisabelga		Wp	Cal	Mas	CH-WI
Tamarindus indica L.	Caesalpiniaceae	Pusga	Ntomi, Toni	Le, Ro, Fr, Mi	Mac, Dec, Tri	Bat, Dri, Fum	EP, MA-MD, HA-CL
Vitellaria paradoxa C.F. Gaertn	Sapotaceae	Taanga	Schi yiri, Si yiri	Le, Ro, Mi	Dec, Tri	Bat, Dri, Fum	MA-MD, CH-WI, HA-CL
Vitex doniana Sweet	Verbenaceae	Aadga	Koto	Le, Ro	Dec	Bat, Dri	MA-MD
Ximenia americana L.	Olacaceae	Leenga		Le, Ro	Dec, Cal	Bat, Dri	MA-MD, CH-WI, HA-CL
Zanthoxylum zanthoxyloïdes Lam. Zep et Timl	Rubiaceae	Rapeoka	Wo	Ro, Ba	Tri	Dri, Fum, Mas	EP, MA-MD, HA-CL, IN
*Zizyphus mauritiana* Lam.	Rhamnaceae	Mugunuga	Tomonon	Le, Ro, Mi	Dec, Tri	Bat, Dri	EP, HA-CL

**Part used:** Whole plants (Wp); Leaves (Le); Stems (St); *Roots (Ro); Barks (Ba);* Flowers (Fl); *Fruits (Fr);* Mistletoes *(Mi); Bulbs (Bu); Latex (La).* **Mode of preparation:** Maceration (Mac); Decoction (Dec); Calcination (Cal); Trituration (Tri). **Mode of administration:** Bath (Bat); Drink (Dri); Fumigation (Fum); Purging (Pur); Massage (Mas); Ingestion (Ing). **Pathologies:** Epilepsy (EP); Madness or Mental Disorders (MA-MD); Charm or Witchcraft (CH-WI); Hallucination or Consciousness Loss (HA-CL); Insomnia (IN); Nerves diseases (ND).

**Table 2:** Main plants used, rate and age of TH user, rate of treated diseases and type of use.

Plants	User TH Rate (%)	Average Age of TH	Treated Diseases Rate (%)	Use Alone or Associated
*Acacia sieberiana DC.*	7.5	45	75	alone
*Afzelia africana Smith ex Pers.*	11.3	42	75	alone, associated
*Allium sativum L.*	20.75	57.5	50	associated
*Annona senegalensis Pers.*	17	55.5	100	alone, associated
*Calotropis procera (Ait) Ait. F.*	20.75	57	100	alone, associated
*Citrus aurantifolia (Christm.) Swingle*	7.5	51	75	associated
*Daniellia oliveri (Rolfe) Hutch. et Dalz.*	19	45.5	75	alone, associated
*Datura innoxia Mill.*	13.2	60.5	75	alone, associated
*Detarium microcarpum Guill. et Perr.*	5.7	39	75	associated
*Diospyros mespiliformis Hochst ex A. DC.*	13.2	39	100	alone, associated
*Ficus iteophylla Miq.*	7.5	39	75	alone, associated
*Guiera senegalensis J.F. Gmel.*	13.2	50	100	alone, associated
*Khaya senegalensis (Desr) A. Juss*	20.75	47	100	alone, associated
*Mitragyna inermis (Willd) O. Ktze*	7.5	35.5	75	alone, associated
*Parkia biglobosa (Jacq.) R. BR. ex G. Don.F*	7.5	48	75	alone
*Securidaca longepedunculata Fresen*	45.3	48	100	alone, associated
*Tamarindus indica L.*	11.3	46	75	associated
*Ximenia americana L.*	5.7	46	75	alone, associated
*Zanthoxylum zanthoxyloïdes Lam. Zep &Timl*	7.5	60	75	alone, associated
*Zizyphus mauritiana Lam.*	5.7	47	75	alone, associated

**Table 3:** Pathologies treated, traditional healers (TH) rate and medicinal plants used.

Pathologies			Treating TH Rate (%)	Number of Plants Used
English Name	Local Name (*Mooré*)	Local Name (*Dioula*)		
Epilepsy	*Kisinkindou*	*Cricromansian*	49	31
Hallucination or Consciousness loss	*Ningyilinga, sobgré*	*Djina bana*	79.2	37
Insomnia	*Gueim Baansé*	*Sinõgõtan ya*	3.8	2
Mental disorders or Madness	*Guimdo, Jougkolgo*	*Faatõ ya*	47.2	32
Nerves diseases	*Guiin Baansé*	*Fassadjourou bana*	3.8	1
Witchcraft or Charm diseases	*Rabsgo, Soondo*	*Soubaga ya*	35.8	25

**Table 4:** Phytochemical constituents and pharmacological properties of main plants used.

Plants	Pharmacological Properties	Phytochemical Constituents	Chemical Compounds Identified
*Acacia sieberiana* DC.	Inhibition of acetylcholinesterase, anti-inflammatory [55].	Alkaloids, cyanogenic glucoside, tannins, terpenoids, Saponins, Flavonoids, essential oils, Cardiac glycosides, steroid, resins [56,57,58].	Dihydroacacipetalin; acacipetalin [56]. Manganese; calcium; magnesium, cupper, iron, zinc, nickel [57].
*Afzelia africana* Smith ex Pers.		Alkaloids, tannins, saponins, fiber, flavonoids, cyanides, beta-carotenes, cyanogenic glycosides, terpenoids, steroids, anthocyanins [59,60,61].	Sodium; potassium; calcium; magnesium; phosphorus; iron; zinc; vitamins A, C, E, B1, B2, B6, B12 [59,62].
*Allium sativum* L.	Stimulant, antioxydant, anti-inflammatory, antimicrobial, fungicidal, antibacterial, anticancerous, chemopreventive, anti-tumoral, antidiabétic [63,64,65].	Alkaloids, phenolics, flavonoids, essential oils [64,66,67]	Trisulphide-di-2-propenyl; artumerone; tetrazolo [1,5-b] pyridazine; 2-hydroxyethyl ethyl disulfide; cyclic octa-atomic sulphur [66]. Alliin; allicin [63]. Diallyl trisulfide; diallyl disulfide; methyl allyl trisulfide [65]. Diallyl monosulfide; trisulfide méthyl-2-propenyl; diméthyl tétrasulfide [68].
*Annona senegalensis* Pers.	Anticonvulsant, anxiolytic, sedative, antibacterial, anti-inflammatory, cytotoxic, antioxydant, anti-nociceptive, antivenenous [15,69].	Alkaloids, flavonoids, saponins, sterols, flavonols, triterpenes, diterpenoids phenols, antraquinones, anthocyanes, coumarines [15,70].	1,2-benzenediol; butylate hydroxytoluene; methylcarbamate; n-hexadecanoique acid; hexadecane; acide oleique; etracosane; 9- octylheptadecane; heneicosane; 13-octadecadien-1-ol; octadecanoique acid; 9,17-octadecandienal; pentadecane; tetratriacontane; squalene [71]. Kaurenoic acid [69]
*Calotropis procera* (Ait) Ait. F.	Anticonvulsant, analgesic, anti-inflammatory,antitumoral, hepatoprotective, antioxidant, spasmolytic, cytotoxifc, cardio-stimulantg, lipase inhibitory, anti-apoptotic [42,72,73,74].	Alkaloids, cardenolides, triterpenes, flavonoids, sterols, saponins, diterpenes, resines, tannins, steroides [43,75].	Calactin; calotropagenin; calotropin; calotoxin; uscharin; syriogenin, afrogenin [42,43]. Flavonoid 5-hydroxy-3,7-dimethoxyflavone-4’-O-β-glucopyranoside; 3-O-rutinosides of quercetin; kaempferol; isorhamnetin [75]. Cholin; uscharin; uscharidin; voruscharidin; α-amyrine; β-amyrine [30,76].
*Citrus aurantifolia* (Christm.) Swingle	Antioxidant, anti-inflammatory, fungicidal, antibactérial [77,78,79].	Essential oils, glucosides, carotenoïds, flavonoids [67,77].	α-pinene; camphene; sabinene; β-pinene; myrcene; ∆3-carene; limonene; (Z)-β-ocimene; α-terpinene; γ-terpinene ; terpinolene; linalool; citronnelal; isocamphene; borneol; terpinen-4-ol; myrtenal; δ-cadinene; caryophyllen oxide; α-eudesmol; myrcene; p-cymene; benzoic acid; α-cedrene; α-bergamotene; α-bisabolene [77,78,79]. Hespéridine, vitamine C [67].
*Daniellia oliveri* (Rolfe) Hutch. et Dalz.	Analgésic, antihistaminic, relaxant, anti-inflammatory, antimicrobial, antidiabetic, antispasmodic, antipyretic, antidiarrhoeal [80,81,82]	Alkaloids, saponosides, flavonoids, glycosides, diterpenoids, sitosterol, coumarines, antracenosides, tanins, hétérosides cardiotoniques, trierpènes, Sterols [8,81,82].	Rutin; quertcitin-3/-O–methyl–3–O-a-rhamnopyranosyl-(→)-β-D-glucopyranoside (Narcissin); quercitrin; quercimeritrin [80,81].
*Datura innoxia* Mill.	Hallucinogen, analgesic, hypnotic, narcotic, anti-cholinergic, antiparkinsonien, sedative, cytotoxic, aphrodisiac, antispasmodic, antiemetic, anti-aflatoxine, anti-bradycardic, anti-inflammatory, anti-dizziness, antitumor [83,84,85]	Alkaloids tropanics [83,86].	Hyoscyamine; scopolamine; tropinone; tropine; pseudotropine; scopoline; scopine; 3-acetoxytropane; 3-acetoxy-6-hydroxytropane; cuscohygrine; aposcopolamine; 3(α’),6-ditigloyloxytropane; 3(β’),6-ditigloyloxytropane; 3-(‘-acetoxytropoyloxy)-tropane; 3,6-Ditigloyloxy-7-hydroxytropane; 7-hydroxyhyoscyamine; 6-hydroxyhyoscyamine; 3-tropoyloxy-6-isovaleroyloxytropane; 6-tigloylhyoscyamine; luteoline [83,85,86].
*Detarium microcarpum* Guill. et Perr.		Alkaloid, fibers, tannins, saponins, flavonoids, cyanides, beta carotenes, cyanogenic glycosides, terpenoids, steroids, anthocyanines [59,61].	Calcium; phosphorus; iron; zinc; vitamins A, E [59].
*Diospyros mespiliformis* Hochst ex A. DC.	Antioxydant, astringent, spasmolytic, antibacterial, homeostatic [87].	Alkaloids, polyphenols, flavonoids, anthraquinones, tannins, triterpenes, saponins, saponosides, anthocyanes, anthracenosides, steroids [87,88].	
*Ficus iteophylla* Miq.	Analgesic, anti-inflammatory, antibacterial [89]	Steroids, furanocoumarines, flavonoids glycosides [80,89]	3β-cholest-5-ene-3, 23diol; 24 ethyl cholest-5-ene- 3β-ol [89].
*Guiera senegalensis* J.F. Gmel.	Psychoactive, detoxicant, anti-plasmodial, antimicrobial, antifungal, antioxydant, anticancerous, antiviral, [90,91].	Alkaloids, flavonoids, triterpenes, tannins, cardenolides, anthracene, coumarines, sterols, saponosides [91,92].	
*Khaya senegalensis* (Desr) A. Juss	Anticonvulsant, Anxiolytic, sedative, antioxydant, anti-tumoral, chemopreventive, anti-inflammatory [15,93,94,95].	Alkaloids, saponins, tannins, triterpenes, flavonoids, glucosides, carbohydrate, phylates, oxalates, triterpenoids [15,94,95].	Gedunin; methyl-angolensate; methyl-6-hydroxyangolensate [96]. Catechin; rutin; quercetin rhamnoside; procyanidins [97]. Fissinolide; 2,6-dihydroxyfissinolide; methyl 3b-acetoxy-6-hydroxy-1-oxomeliac-14-enoate [98]. Magnesium, calcium, potassium, sodium, zinc, iron, manganese, lead, chromium [94].
*Mitragyna inermis* (Willd) O. Ktze	Anticonvulsant, cardiovascular affects, antibactérial, antiplasmodial, anti-diabetic [99,100,101].	Alkaloids, polyphenols, sterols, polyterpenes, quinones, tannins, saponins, flavonoids, saponosides [99,100,102].	Rhynchophylline; isorhynchophylline; corynoxeine; isocorynoxeine; ciliaphylline; rhynchociline; isospcionoxeine; 9-methoxy-3-epi-α-yohimbine [103]. 27-nor-terpenoid glucoside [104,105].
*Parkia biglobosa* (Jacq.) R. BR. ex G. Don. F	Antibacterial, antifungal, antioxidant, antihyperlipidemic, cardioprotective [106,107,108].	Alkaloids, cardiac glycosides, tannins, steroids, tannins, alkaloids, flavonoids, saponins, terpenes, glycosides [106,109].	
*Securidaca longepedunculata* Fresen	Anticonvulsant; antidepressant, anxiolytic, antioxydant, anti-nociceptive, cytotoxic, antivenomous, antibactérial, aphrodisiac, sedative, [110,111,112]	Alkaloids, saponosides, flavonoids, phenols, xanthones, anthraquinones, essential oils [113,114,115].	Gallic acide; quercetin; cafeic acid; chlorogenic acid; epicatechin; p-coumaric acid; cinnamic acid; rutin; apigenin [82] Phelandrene; pinene; z-sabinol; limonene; p-cymene [110] Securinin [116,117]. Muchimangine E, muchimangine F [118].
*Tamarindus indica* L.	Analgesic, antinociceptive, antivenin, hepatoprotective, anti-inflamatory, anti-helminthic, antioxydant, antibacterial [119,120,121].	Alkaloids, saponins, glycosides, tannins, terpenoids, flavonoids, coumarins, naphthoquinones, anthraquinones, xanthonones [121,122,123,124].	C-glycosidesorientin; vitexin; isoorientin; isovitexin; tartaric acid; malic acid [120]. Limonene; methyl salicylate; pyrazine; alkylthiazole; calcium; iron; zinc; vitamins B and C [125].
*Ximenia americana* L.	Anti-plasmodiale, antioxidant, anticancer, antineoplastic, antitrypanosomal, antirheumatic, antioxidant, analgesic antipyretic [90,126,127].	Alkaloids, anthraquinones, cardiac glycosides, flavonoids, pylobatannins, saponnins, tannins, terpenoids, isoprenoids, triterpenes, sesquiterpenes, quinones [126,127,128].	Norisoprenoid isophorane; ximenynic acid; methyl-14,14-dimethyl-18-hydroxyheptatracont-27,35-dienoate; dimethyl-5-Methyl-28,29-dihydroxydotriacont-3,14,26-triendioate; 10Z,14E,16E-octadeca-10,14,16-triene-12-ynoic acid, tariric acid; β-sitosterol; oleanene palmitates [127,129,130] .
*Zanthoxylum zanthoxyloïdes* Lam. Zep &Timl	Antiplasmodial, vasorelaxant, antifungal, antibacterial, inhibition of acetylcholinesterase, antiradical, [131,132,133].	Alkaloids, tannins [132,134].	Myrcene; germacrene D; limonene, β-caryophyllene; decanal [135]. Acide 3,4-O-divanilloylquinique, acide 3,5-O-divanilloylquinique, acide 4,5-O-divanilloylquinique [136].fagaramide; (+)-sésamine; lupéol; hespéridine; Dihydrochélérythrine; N,N-diméthyllindcarpine; Chélérythrine; Norchélérythrine; 6-(2-oxybutyl) dihydrochélérythrine; 6-hydroxy-dihydrochélérythrine; avicine; arnottianamide [131] .
*Zizyphus mauritiana* Lam.	Antitumor, antibacterial, antioxidant, antimicrobial, anticancer [137,138].	Alkaloids, flavonoids, triterpenoids, tannins, glycoside, phenol, lignin, saponins [137,139].	2H-1-benzopyran-2-one; 9, stigmasterol; stigmastane-3,6-dione [137]. 3-methyl piperidine; o-methyl delta-tochopherol; octacosane; cyclobarbital; squalene; 2,4-dimethyl; thymol TMS; benzoquinoline; γ-sitosterol; hydroprogesterone [138].
